# The serum level of vitamin D and prevalence of vitamin D deficiency among children with asthma in Asia and Africa: a systematic review and meta-analysis

**DOI:** 10.1186/s13690-024-01321-5

**Published:** 2024-07-05

**Authors:** Ermias Sisay Chanie, Guicheng Zhang, Peter Le Souef

**Affiliations:** 1 Department of Paediatric and Child Health, Debre Tabor University, Debre Tabor, Ethiopia; 2 School of Population Health, Curtin University, Perth, WA 6102 Australia; 3 School of Medicine, The University of Western Australia, Perth, WA 6008 Australia

**Keywords:** Children, Asthma, Vitamin D, Vitamin D deficiency, Asia and Africa

## Abstract

**Background:**

Several studies on the serum level of vitamin D and the percentage of vitamin D deficiency in children with asthma have been conducted in Asia and Africa, but the results have been inconsistent and inconclusive, requiring a systematic review and meta-analysis to assess the strength of the evidence.

**Objective:**

The objective of this review is to synthesize evidence on serum levels of vitamin D and the percentage of vitamin D deficiency among children with asthma in Asia and Africa**.**

**Methods:**

To identify relevant articles, a comprehensive search was conducted across various databases and repositories such as PubMed, Google Scholar, Hinary, Web of Science, ResearchGate, as well as gray literature sources. The Preferred Reporting Items for Systematic Review and Meta-Analysis (PRISMA) guidelines were followed during the retrieval process. Data extraction was performed following a standardized format based on the JBI (Joanna Briggs Institute) data extraction guidelines. Microsoft Excel was utilized for data extraction, and subsequently, the data was exported to STATA 17 for further analysis. To assess the heterogeneity among the included studies, Cochrane Q-statistics and the I2 tests were employed. Publication bias was assessed using the Egger test and funnel plot.

**Result:**

This meta-analysis investigated 33 articles encompassing a total of 3432 children diagnosed with asthma. The findings demonstrated that in low- or middle-income countries across Africa and Asia, children with asthma had an average serum vitamin D level of 21.9 ng/ml (95% confidence interval [CI]: 18.0–25.9 ng/ml), with 53.7% (95% CI: 40.5–66.9) experiencing vitamin D deficiency. Additionally, when considering the continent, children with asthma in Asia had an average serum vitamin D level of 18.5 ng/ml (95% CI: 13.8–23.3), while those in Africa had a level of 28.7 ng/ml (95% CI: 22.7–34.8). The analysis further explored different sub-group analyses. Depending on the study design, case–control studies yielded an average serum vitamin D level of 20.3 ng/ml (95% CI: 18.2–22.4), whereas cross-sectional studies resulted in 23.8 ng/ml (95% CI: 17.5–30.1). Based on publication year, studies published on or before 2015 had an average serum level of 21.0 ng/ml (95% CI: 18.0–24.0), while those published after 2015 had a level of 22.4 ng/ml (95% CI: 17.2–27.7). Moreover, when considering sample size, studies with 100 participants or less had an average serum level of 21.7 ng/ml (95% CI: 17.3–26.1), while studies with more than 100 participants had a level of 22.1 ng/ml (95% CI: 18.6–25.6).

**Conclusion:**

Children with asthma in Asia and Africa were found to have low serum levels of vitamin D and a high percentage of vitamin D deficiency. This highlights the importance of early detection and monitoring of vitamin D levels in these children to prevent potential complications associated with vitamin D deficiency. Taking proactive measures to address and manage vitamin D deficiency is crucial for the well-being of children with asthma in these regions.

**Table:** 

Text box 1. Contributions to the literature
• This systematic review and meta-analysis critically examine existing literature on vitamin D levels and the prevalence of deficiency among children with asthma in Asia and Africa, contributing to our understanding of how vitamin D deficiency impacts this specific population globally.
• Through identifying geographical variations in vitamin D deficiency among children with asthma in Asia and Africa, this research expands our knowledge and facilitates the implementation of targeted public health strategies and interventions aimed at optimizing vitamin D status and improving asthma management in these specific regions.
• Through identifying geographical variations in vitamin D deficiency among children with asthma in Asia and Africa, this research expands our knowledge and facilitates the implementation of targeted public health strategies and interventions aimed at optimizing vitamin D status and improving asthma management in these specific regions.
• By contextualizing the study's findings within the broader body of research on vitamin D deficiency and asthma, this research underscores the significance of considering vitamin D status as a modifiable factor in the prevention and management of asthma among children in Asia and Africa. These insights contribute to ongoing efforts to enhance respiratory health outcomes in these regions.

## Introduction

Asthma, a prevalent chronic lung disease in children and adults [1, 2], causes symptoms like coughing, wheezing, and breathing difficulties due to narrowed airways [1, 3]. The risk factors for asthma include having a family history, respiratory infections, exposure to air pollution, smoking, allergies, and weather condition [1, 3, 4]. Children with asthma commonly experience sleep problems, recurrent respiratory infections, fatigue, learning disabilities, and growth retardation [2, 5, 6]. If left untreated or poorly managed, childhood asthma can lead to complications such as obstructive sleep apnea, anxiety, pneumonia, and even respiratory failure [6–8].

Asthma is a significant global health issue, with a considerable number of deaths and a high prevalence of affected individuals. In 2019, asthma was responsible for 455,000 deaths, impacting an estimated 262 million people [1]. Particularly in Africa, there has been a substantial increase in the number of children with asthma between 1990 and 2010, there were 34.1 million and 49.7 million more children with asthma in Africa [9], and asthma is also becoming more prevalent in Asia [10].

Vitamin D has been increasingly associated with the development and management of asthma. It plays a role in activating specific immune cells that regulate the severity and frequency of asthma attacks [11–13]. Additionally, higher vitamin D levels have been linked to reduced inflammation markers and improved control of asthma symptoms [12, 14, 15]. However, many children with asthma have low levels of vitamin D, which may lead to more frequent asthma attacks and increased reliance on medications [14, 16]. The normal vitamin D level for children is considered to be equal to or greater than 50 nmol/L [17], but most children with asthma have been found to have levels below 20 ng/ml [14, 18, 19]. These levels are categorized as insufficient (20 to 30 ng/ml) or deficient (< 20 ng/ml) according to reference laboratories [20, 21].

Asthma and vitamin D deficiency often coexist and can have a reciprocal negative impact on each other [12, 16]. Children with asthma are more likely to have vitamin D insufficiency or deficiency, which is associated with increased asthma symptoms, exacerbations, decreased lung function, higher medication use, and more severe disease [22, 23]. The prevalence of vitamin D deficiency in children with asthma varies widely, ranging from 28.5% to 90.6% [24, 25], with mean values ranging from 9.0 ng/ml to 68.6 ng/ml [26, 27].

While several studies have explored the relationship between vitamin D levels and asthma in children from Africa and Asia, the findings have been inconsistent and inconclusive. Hence, the scope of this study is to conduct a systematic review and meta-analysis focused on examining the serum levels of vitamin D and the percentage of vitamin D deficiency among children with asthma specifically in the Asian and African continents. The study aims to synthesize and analyze existing evidence from relevant studies conducted in these regions to provide a comprehensive understanding of the relationship between vitamin D status and asthma in children. Additionally, the study seeks to explore potential variations across different populations and evaluate the impact of vitamin D levels on asthma outcomes among children in Asia and Africa.

## Methods

### Design and search strategy

#### Review question

This systematic review and meta-analysis aimed to answer two main questions: What are the vitamin D serum levels among children with asthma in Africa and Asia? and what is the prevalence of vitamin D deficiency among children with asthma in Africa and Asia?

#### Reporting

The review protocol has been sent to the PROSPERO database for registration with ID 422317. The systematic review and meta-analysis were reported using the Preferred Reporting Items for Systematic Reviews and Meta-Analyses (PRISMA) [28] statement guideline to determine the serum level of vitamin D and percentage of vitamin D deficiency among children with asthma in African. A systematic search was conducted across various international databases, such as PubMed, Google Scholar, Hinary, Web of Science, ResearchGate, as well as gray literature sources (Table 1).

**Table 1:** The Search strategy of the serum level of vitamin D and the percentage of vitamin D deficiency among children with asthma in Asia and Africa

#1	("serum level of vitamin D" OR "vitamin D levels" OR "vitamin D concentration" OR "vitamin D percentage" OR "vitamin D prevalence" OR "vitamin D magnitude" OR "vitamin D epidemiology OR "vitamin D deficiency)
#2	("children” OR " Kids” OR " infant” OR " adolescents")
#3	(Africa" OR Low-income countries " OR "Developing nations " OR " Resource-constrained settings " OR "economically disadvantaged countries " OR " Asia)
#4	Search #1 AND #2 AND #3 not animal studies
#5	Search links: https://pubmed.ncbi.nlm.nih.gov/?term=%28%22serum+level+of+vitamin+D%22+OR+%22vitamin+D+levels%22+OR+%22vitamin+D+concentration%22+OR+%22vitamin+D+percentage%22+OR+%22vitamin+D+prevalence%22+OR+%22vitamin+D+magnitude%22+OR+%22vitamin+D+epidemiology+OR+%22vitamin+D+deficiency%29+AND+%28%22children%E2%80%9D+OR+%22+Kids%E2%80%9D+OR+%22+infant%E2%80%9D+OR+%22+adolescents%22%29+AND+%28Africa%22+OR+Low-income+countries+%22+OR+%22Developing+nations+%22+OR+%22+Resource-constrained+settings+%22+OR+%22economically+disadvantaged+countries+%22+OR+%22+Asia%29

## Characteristics of the included articles

### Eligibility criteria

#### Inclusion criteria

##### Study area

Studies conducted on the Asia and African.

##### Publication conditions

Articles published in peer-reviewed journals and articles available on the university website.

##### Study design

All observational study designs (cross-sectional and case–control) reporting the serum level of vitamin D and/or percentage of vitamin D deficiency among children with asthma in Asia and Africa countries were considered.

##### Outcome of interests

Studies reported data on the serum level of vitamin D and percentage of vitamin D deficiency among children with asthma in Asia and/or Africa were considered in the Population, Exposure, Comparison, and Outcome (PICO) format.

##### Language

Articles reported in the English language were considered.

### Exclusion criteria

Articles, which were not fully accessed, were excluded because of inability to assess the quality of articles in the absence of full text and to estimate the outcome variable. Moreover, case reports, case series, and studies not differentiating asthma children from other respiratory disorders were excluded from the study.

### Outcome measurement among children with asthma

This systematic review has two main outcome variables. The primary outcome variable of this study is a percentage of vitamin D deficiency. The percentage of vitamin D deficiency was calculated from each primary study by dividing the number of vitamin D deficiency with asthma by the total number of children with asthma multiplied by 100 [29, 30]. The second outcome was to determine the serum level of vitamin D [30, 31]. According to the criteria established in this study, children are defined as individuals who are younger than 18 years of age.

### Data extraction

All titles and abstracts were exhaustively screened through using a standardized JBI data extraction format to extract all potentially relevant data. The primary author, continent/country, year publication, study area, study design, sample size, and serum level of vitamin D with 95% confidence intervals or with the standard deviation score of each study were included in the data extraction format.

Further information and differences of opinion were settled by discussion and agreement at the time of data abstraction.

The retrieved studies were exported to the citation manager (Zotero) and then duplicate articles were excluded. Disagreements were discussed during a consensus meeting with other reviewers for the final selection of studies to be included in the systematic review and meta-analysis.

### Quality assessment

The Joanna Briggs Institute Critical Appraisal tool for use in JBI Systematic Reviews (JBI-MAStARI) was used to assess the trustworthiness, relevance, and results of published papers [32]. The tool compromises eight main standards for evaluating each primary study critically. As a result, primary studies that had a score of 50% or higher were included in the systematic review and meta-analysis study.

### Statistical analysis

The extracted data were edited, cleaned, and checked for completeness in a Microsoft Excel sheet, and then exported into STATA 17 for further analysis. Pooled overall serum level of vitamin D and percentage of vitamin D deficiency among children with asthma were estimated through a random effect meta-analysis model. Cochrane Q-statistics and the I2 test's *p*-values were used to assess the heterogeneity of reported serum levels of vitamin D and the percentage of vitamin D deficiency among studies [33, 34].

The publication bias was estimated through the Egger test and funnel plots [35] and sensitivity analysis was piloted to examine the effect of a single study on the overall estimation. Moreover, subgroup analysis was performed by the study continent, study design, sample size, and year of publication.

## Results

Out of the initial pool of 1750 studies, after removing duplicates, 577 studies remained. Among these, 434 studies were excluded based on the assessment of their titles, abstracts, and study nature (such as case reports and qualitative studies). After this exclusion, 143 full-text articles were assessed, and 110 of them were further excluded as they did not report the outcome variable of interest. finally, a total of 33 studies with full-text articles were included in the review. These studies involved a combined sample size of 3,432 participants. The studies were conducted in 12 different countries across Africa and Asia, including Egypt, Turkey, Nigeria, Iran, China, India, South Korea, Tunisia, Israel, Iraq, Thailand, and Qatar. (Fig. S1).

### Characteristics of the included articles

A total of 3,432 asthmatic children from 12 countries in Asia and Africa were included in the 33 studies comprising this meta-analysis, published between 2011 and 2022.

From a total of 33 studies, 12 studies were reported from Egypt [36–41], and Turkey [25, 42–46]. Additionally, 15 were reported from Nigeria [27, 47–49], Iran [20, 50–52], China [21, 53–55], and India [26, 56, 57]. Furthermore, 6 studies were reported from South Korea [58], Tunisia [59], Israel [60], Iraq [61], Thailand [62], and Qatar [63].

From the total of 33 studies, 16 studies were cross sectional [25–27, 39, 40, 44–47, 49, 50, 52, 58, 60–62], and 17 were case–control studies [20, 21, 37, 38, 41–43, 48, 51, 53–57, 59, 63, 64].

All of the studies included in this review were observational stuies, with a sample size ranging from 36 participants reported from a study in Egypt [64], and 483 from Qatar [63].

Of the 33 studies, 27 studies described both the serum level of vitamin D and the percentage of vitamin D deficiency, whereas, 6 studies only reported the serum level of vitamin D in children with asthma in Africa and Asia continents (Table 2).

**Table 2:** The serum level of vitamin D and percentage of vitamin D deficiency distribution among children with asthma in Asia and Africa

First Author/year	Country	Continent	Study Design	Sample size	Mean value of vitamin D	Vitamin D deficiency (%)	Quality status
**Nabih et al. (2014) ** [38]	**Egypt**	**Africa**	**Case–control**	**180**	**26 ± 12.5**	**77.3**	**Low risk**
**Kuti et al. (2021)** [48]	**Nigera**	**Africa**	**Case–control**	**90**	**38 ± 17**	**26**	**Low risk**
**Ibegbu et al. (2022)** [27]	**Nigera**	**Africa**	**Cross sectional**	**65**	**68.6 ± 25.9**	**1.5**	**High risk**
**Elnadya et al. (2013) ** [40]	**Egypt**	**Africa**	**Cross sectional**	**50**	**24.1 ± 2.9**	**40**	**Low risk**
**Omole et al. (2018) ** [49]	**Nigera**	**Africa**	**Cross sectional**	**103**	**49.2 ± 7.2**	**2**	**High risk**
**Gamal et al. (2018) ** [39]	**Egypt**	**Africa**	**Cross sectional**	**90**	**12.2 ± 1.9**	**80**	**Low risk**
**El-Menem et al. (2013)** [41]	**Egypt**	**Africa**	**Case–control**	**60**	**39 ± 12**	**45**	**Low risk**
**Ahmed et al. (2020)** [37]	**Egypt**	**Africa**	**Case–control**	**36**	**7.69 ± 2.3**	**75**	**Low risk**
**Kim (2017) ** [58]	**S/Korea**	**Asia**	**Cross sectional**	**50**	**16.3 ± 4.2**	**86**	**Low risk**
**Mohammad et al. (2020) ** [50]	**Iran**	**Asia**	**Cross sectional**	**200**	**20.4 ± 13.4**	**36.5**	**Low risk**
**Ahmed et al. (2020) ** [37]	**Egypt**	**Africa**	**Case–control**	**50**	**13.5 ± 10.5**	**72**	**Low risk**
**KAVITHA et al. (2017) ** [26]	**India**	**Asia**	**Cross sectional**	**105**	**9 ± 5.1**	**66.7**	**Low risk**
**Maalmi (2013) ** [59]	**Tunisia**	**Africa**	**Case–control**	**155**	**18.9 ± 6.7**	**61.3**	**Low risk**
**Zhang et al. (2017) ** [53]	**China**	**Asia**	**Case–control**	**143**	**22.2 ± 10.5**	**48.9**	**Low risk**
**Dabbah et al. (2015) ** [60]	**Israel**	**Asia**	**Cross sectional**	**71**	**23 ± 7.7**	**36.6**	**Low risk**
**Uysalol et al. (2013) ** [25]	**Turkey**	**Asia**	**Cross sectional**	**85**	**16.6 ± 8.5**	**29.4**	**Low risk**
**Anand et al. (2021) ** [56]	**India**	**Asia**	**Case–control**	**101**	**17.3 ± 12.2**	**64.4**	**Low risk**
**Dogru (2014) ** [42]	**Turkey**	**Asia**	**Case–control**	**120**	**21.5 ± 7.7**	**43.3**	**Low risk**
**Kang et al. (2018)** [54]	**China**	**Asia**	**Case–control**	**96**	**18.9 ± 3.6**	**Not found**	**Low risk**
**Hou et al. (2018) ** [55]	**China**	**Asia**	**Case–control**	**70**	**16.8 ± 3.3**	**Not found**	**Low risk**
**Yusuff et al. (2022) ** [47]	**Nigera**	**Africa**	**Cross sectional**	**128**	**22.4 ± 2.9**	**Not found**	**Low risk**
**Al-Sharifi et al. (2017) ** [61]	**Iraq**	**Asia**	**Cross sectional**	**50**	**2.05 ± 0.4**	**Not found**	**Low risk**
**Bai and Dai (2018) ** [21]	**China**	**Asia**	**Case–control**	**57**	**17.5 ± 2.1**	**Not found**	**Low risk**
**Thomas et al. (2019) ** [45]	**Turkey**	**Asia**	**Cross sectional**	**73**	**12.1 ± 7.4**	**82.2**	**Low risk**
**Havan et al. (2017) ** [44]	**Turkey**	**Asia**	**Cross sectional**	**72**	**14.2 ± 6.7**	**80.6**	**Low risk**
**Hatami et al. (2014) ** [51]	**Iran**	**Asia**	**Case–control**	**200**	**20.3 ± 2.8**	**56**	**Low risk**
**Awasthi (2014) ** [57]	**India**	**Asia**	**Case–control**	**50**	**20.8 ± 8.1**	**57.1**	**Low risk**
**Krobtrakul et al. (2013) ** [62]	**Thailand**	**Asia**	**Cross sectional**	**125**	**25.9 ± 9.4**	**19.2**	**Low risk**
**Alyasin et al. (2011) ** [52]	**Iran**	**Asia**	**Cross sectional**	**50**	**49.3 ± 21.4**	**4**	**High risk**
**Kilic et al. (2019) ** [43]	**Turkey**	**Asia**	**Case–control**	**100**	**22.2 ± 12.3**	**58.4**	**Low risk**
**Esfandiar et al. (2016) ** [20]	**Iran**	**Asia**	**Case–control**	**53**	**14.5 ± 8.1**	**73.6**	**Low risk**
**Bener et al. (2012) ** [63]	**Qatar**	**Asia**	**Case–control**	**483**	**17.2 ± 11.1**	**68.1**	**Low risk**
**Ozdogan et al. (2017) ** [46]	**Turkey**	**Asia**	**Cross sectional**	**71**	**11.8 ± 10.3**	**4**	**High risk**

### Meta-analysis

The pooled estimation of serum levels of vitamin D and the percentage of vitamin D deficiency among children with asthma in Asia and Africa was derived based on the following studies: Thirty-three studies (*n* = 33) provided data on serum levels of vitamin D, and twenty-seven (*n* = 27) studies reported the percentage of vitamin D deficiency. The serum level of vitamin D ranged from the highest reported level in Nigeria, 68.6 ± 25.9 ng/ml [27], to the lowest reported level in Iraq, 2.05 ± 0.4 ng/ml [61].

Moreover, the percentage of vitamin D deficiency ranged from the highest reported from a study in Turkey 82.2% [45], to the lowest report was from Nigeria 1.5% [27].

The pooled serum level of vitamin D among children with asthma in Africa and Asia was found to be 21.9 ng/ml (95%CI; 18.0–25.9 ng/ml), as displayed in (Fig. S2).

Moreover, the percentage of vitamin D deficiency children with asthma in Africa and Asia was found to be 53.7% (95%CI; 40.5–66.9) (Table 3).

**Table 3:** The pooled percentage of vitamin D deficiency among children with asthma in Africa and Asia

Study	Effect size	[95% conf. interval]		% weight
**Alyasin etal, 2011 ** [52]	4.0	-1.4	9.4	3.8
**Bener et al. 2012 ** [63]	68.1	63.9	72.3	3.8
**Elnadya et al. 2013 ** [40]	40.0	26.4	53.6	3.6
**Krobtrakulchai et al. 2013 ** [62]	19.2	12.3	26.1	3.7
**Maalmi, 2013 ** [59]	61.3	53.6	69.0	3.7
**Uysalol et al. 2013 ** [25]	90.6	84.4	96.8	3.7
**Hatami et al. 2014 ** [51]	56.0	49.1	62.9	3.7
**Nabih et al. 2014 ** [38]	77.3	71.2	83.4	3.7
**Awasthi and Vikram, 2014 ** [57]	57.1	43.4	70.8	3.6
**Dogru, 2014 ** [42]	43.3	34.4	52.2	3.7
**Dabbah et al. 2015 ** [60]	36.6	25.4	47.8	3.7
**Ahmed et al. (2020)** [37]	75.0	60.9	89.1	3.6
**El-Menem et al. 2013** [41]	45.0	32.4	57.6	3.7
**Esfandiar et al. 2016 ** [20]	73.6	61.7	85.5	3.7
**Havan et al. 2017 ** [44]	80.6	71.5	89.7	3.7
**Kim, 2017 ** [58]	86.0	76.4	95.6	3.7
**Zhang et al. 2017 ** [53]	48.9	40.7	57.1	3.7
**KAVITHA et al. 2017 ** [26]	66.7	57.7	75.7	3.7
**Gamal et al. 2018 ** [39]	80.0	71.7	88.3	3.7
**Omole et al. 2018 ** [49]	2.0	-0.7	4.7	3.8
**Kilic et al. 2019 ** [43]	58.4	48.7	68.1	3.7
**Thomas et al. 2019 ** [45]	82.2	73.4	91.0	3.7
**Mohammadzadeh et al. 2020 ** [50]	36.5	29.8	43.2	3.7
**Kuti et al. 2021** [48]	26.0	16.9	35.1	3.7
**Ahmed et al. 2020 ** [37]	72.0	59.6	84.4	3.7
**Anand et al. 2021 ** [56]	64.4	55.1	73.7	3.7
**Ibegbu et al. 2022** [27]	1.5	-1.5	4.5	3.8
**Theta**	53.702	40.531	66.872	

Number of studies = 27, Heterogeneity: tau^2^ = 1.2e + 03, I^2^ (%) = 99.04, H^2^ = 103.90 Test of theta = 0: z = 7.99, Prob >|z|= 0.0000, Test of homogeneity: Q = chi2 (26) = 2701.40, Prob > Q = 0.0000

### Sub group analysis of pooled of serum levels of vitamin D among children with asthma in Africa and Asia

The subgroup analysis was employed for a dual purpose, as it not only estimates the pooled mean within each group but also tests for differences in the pooled means between groups for deeper into the subject matter to ensure a comprehensive understanding.

In this regard, the studies were stratified by continent, study design, year of publication, and sample size. The pooled serum level of vitamin D among children with asthma by continent in Asia and Africa was found to be 18.5 ng/ml (95%CI; 13.8- 23.3) and 28.7 ng/ml (95%CI; 22.7–34.8), by study design of case control and cross-sectional was found to be 20.3 ng/ml (95%CI; 18.2–22.4) and 23.8 ng/ml (95%CI; 17.5–30.1), by year of publication ≤ 2015 and > 2015 was found 21.0 ng/ml (95%CI; 18.0–24.0) and 22.4 ng/ml (95%CI; 17.2–27.7), and by sample size ≤ 100 and > 100 was found to be 21.7 ng/ml (95%CI; 17.3–26.1) and 22.1 ng/ml (95%CI; 18.6–25.6)respectively (Fig. S3-6).

### Sub group analysis of pooled percentage of vitamin D among children with asthma in Asia and Africa

The subgroup analysis was employed to estimate percentage of vitamin D by stratifying the studies into different categories. In this regard, the studies were stratified by continent, study design, year of publication, and sample size. The pooled percentage of vitamin D among children with asthma by continent in Asia and Africa was found to be 57.1%(95%CI; 44.1–70.1) and 47.8%(95%CI; 26.5–69.2), by study design of case control and cross-sectional was found to be 60.8%(95%CI; 53.2–68.3) and 44.9%(95%CI; 25.-64.6), by year of publication ≤ 2015 and > 2015 was found 55.2%(95%CI; 40.1–70.3) and 52.0% (95%CI; 33.3–70.8) and by sample size ≤ 100 and > 100 was found to be 56.7%(95%CI; 36.7–76.7), and 49.2%(95%CI; 29.7–69.0) (Table 4) respectively.

**Table 4:** Summary of subgroup of vitamin D deficiency percentage among children with asthma by continent, study design, year of publication, and sample size in Asia and Africa

Variable	Characteristics	Effect	[95% Conf. Interval]		% Weight
**By continent**	Asia	57.1	44.1	70.1	63.1
	Africa	47.8	26.5	69.2	36.9
**By study design**	Case–control	60.8	53.2	68.3	55.4
	Cross-sectional	44.9	25.1	64.6	44.6
**By year of publication**	≤ 2015	55.2	40.1	70.3	51.7
	> 2015	52.0	33.3	70.8	48.3
**By sample size**	≤ 100	56.7	36.7	76.7	58.9
	> 100	49.2	29.7	69.0	41.0

### Publication bias

Egger's test was used to explain the visual for detecting bias or systematic heterogeneity of serum level of vitamin D and percentage of vitamin D among children with asthma in Asian and African (Table 5). Moreover, the funnel plot also determines the publication bias of serum level of vitamin D and percentage of vitamin D among children with asthma in Asian and African (Fig. S7-8).

**Table 5:** Egger's test of serum level of vitamin D among children with asthma in Asia and Africa

Std_Eff Coefficient		Std. errs	T	*P* > t	[95% conf interval]	
**Slop**e	2.7	1.2	2.1	0.04	0.12	5.2
**bias**	27.1	4.6	5.9	0.0	17.7	36.5

Test of H0: no small-study effects *P* = 0.000 Number studies = 33, Root MSE = 21.45

Furthermore, the results of sensitivity analyses were also examined of serum level of vitamin D and percentage of vitamin D among children with asthma in Asia and Africa respectively (Table 6 and Fig. S9).

**Table 6:** The sensitivity test of serum level of vitamin D among children with asthma in Asia and Africa

Study omitted	Estimate	[95% Conf. Interval]	
**Alyasin et al. (2011) ** [52]	21.1	17.2	25.1
**Bener et al. (2012) ** [63]	22.1	18.1	26.1
**Elnadya et al. (2013) ** [40]	21.9	17.9	25.8
**Krobtrakulchai et al. (2013) ** [62]	21.8	17.8	25.8
**Maalmi (2013) ** [59]	22.0	18.0	26.0
**Uysalol et al. (2013) ** [25]	22.1	18.1	26.1
**Hatami et al. (2014) ** [51]	21.9	18.0	25.9
**Nabih et al. (2014) ** [38]	21.8	17.8	25.8
**Awasthi and Vikram (2014) ** [57]	21.9	17.9	25.9
**Dogru (2014) ** [42]	21.9	17.9	25.9
**Dabbah et al. (2015) ** [60]	21.9	17.9	25.9
**Ahmed et al. (2020)** [37]	22.4	18.3	26.5
**El-Menem et al. (2013)** [41]	21.4	17.5	25.4
**Esfandiar et al. (2016) ** [20]	22.2	18.2	26.2
**Havan et al. (2017) ** [44]	22.2	18.2	26.2
**Al-Sharifi et al. (2017) ** [61]	22.3	19.0	24.6
**Ozdogan et al. (2017) ** [46]	22.3	18.3	26.2
**Kang et al. (2018)** [54]	22.0	18.0	26
**Kim (2017) ** [58]	22.1	18.1	26.1
**Zhang et al. (2017) ** [53]	21.9	17.9	25.9
**KAVITHA et al. (2017) ** [26]	22.3	18.3	26.4
**Bai and Dai (2018) ** [21]	22.1	18.0	26.1
**Gamal et al. (2018) ** [39]	22.3	17.9	26.5
**Omole et al. (2018) ** [49]	21.1	17.3	24.8
**Hou et al. (2018) ** [55]	22.1	18.1	26.1
**Kilic et al. (2019) ** [43]	21.9	17.9	25.9
**Thomas et al. (2019) ** [45]	22.2	18.3	26.2
**Mohammadzadeh et al. (2020) ** [50]	21.9	18.0	25.9
**Kuti et al. (2021)** [48]	21.4	17.5	25.4
**Ahmed et al. (2020) ** [37]	22.2	18.2	26.2
**Anand et al. (2021) ** [56]	22.1	18.1	26.1
**Ibegbu et al. (2022)** [27]	20.6	16.6	24.5
**Yusuff et al. (2022) ** [47]	21	17.9	25.8
**Combined**	21.9	18.0	25.9

## Discussion

This study aims to estimate the pooled serum level of vitamin D and the percentage of vitamin D deficiency among children with asthma in the Asian and African.

In this regard, the pooled serum level of vitamin D and the percentage of vitamin D deficiency was found to be 21.9 ng/ml (95%CI; 18.0–25.9 ng/ml), and 53.7% (95%CI; 40.5–66.9) among children residing in low- or middle-income countries in Africa and Asia respectively.

The serum level of vitamin D among children with asthma in this review was consistent or lower than the study conducted in Russia [65], Mexico [66], UK [67], Italy [68], and U.S.A [69]. Moreover, the proportion of vitamin Deficiency in this study is higher than the study conducted in Spain [70], in U.S.A [69], and in Mexico [66].

This difference might be this review was conducted in a resource-limited setting whereas the above studies were conducted in developed countries since in developed countries the health care provider, patients, and stakeholders may have good equipment, knowledge, and quality of care to reduce and prevent the deficiency of vitamin D among children with asthma. Moreover, supplementation of vitamin D and early detection might be higher in developed countries as compared with a resource-limited setting [71–73].

However, the percentage of vitamin D deficiency among children with asthma in this study is lower than several other studies conducted in Brazil [74], Russia [65], and UK [23]. This discrepancy might be the cut value difference in defining vitamin D deficiency, For example, the cut of vitamin D deficiency in the study conducted in the UK was < 50 nmol/l whereas in this study was < 20 nmol/l [23]. Moreover, the study participant difference or the level of severity/attack or recurrent wheezing might be contributing to this difference [74].

The findings in this review will fill an evidence gap on the average mean value of vitamin D and the extent of vitamin D deficiency among children with asthma in resource-limited settings (i.e. Asia and Africa). Moreover, the review findings illuminate the high prevalence of vitamin D deficiency among children with asthma, offering valuable insights to stakeholders, including non-governmental and governmental organizations, and researchers in the field of childhood asthma. These insights can help focus attention and efforts on mitigating the burden associated with this issue. Given the frequent co-occurrence of asthma and vitamin D deficiency, it is important to address both conditions as they exacerbate each other. [12, 16]. The majority of asthmatic infants have low vitamin D levels, which could make asthma attacks frequent, and severe, and increase the demand for drugs [14, 16].

This study is the estimate the pooled serum level of vitamin D and the percentage of vitamin D deficiency among children with asthma to recognize the issue in general. In addition to that, the study also perform a sub group analysis to see the serum level of vitamin D and the percentage of vitamin D deficiency between the group, it allows researchers to test their hypotheses in more detail, and It is important to understand the risks associated with this kind of analysis of serum level of vitamin D associated with childhood asthma [75–77]. Given the significance of a large sample size for accuracy, the reported pooled serum level of vitamin D and the percentage of vitamin D deficiency among children with asthma in Asia and Africa may potentially be underestimated in this study. Moreover, recent studies focusing on this specific population in the region have indicated a decline in vitamin D levels and an increase in the prevalence of vitamin D deficiency among children with asthma. To avoid drawing misleading conclusions influenced by confounding factors, it is crucial to take into account the subgroup analysis findings presented in this study. These findings highlight the variability in serum vitamin D levels and deficiency rates across different subgroups based on factors such as continent, study design, year of publication, and sample size. Considering these factors will contribute to a more accurate understanding of the actual situation.

### Limitations of the study

While this systematic review and meta-analysis approach multiple studies to estimate the prevalence of vitamin D deficiency and its impact on asthma outcomes in children from Asia and Africa, it is important to acknowledge a limitation. This review did not specifically assess the factors influencing serum levels of vitamin D and the percentage of vitamin D deficiency among children with asthma. Consequently, further research is needed to investigate these factors and gain a more comprehensive understanding of their role in the relationship between vitamin D status and asthma outcomes in this population.

## Conclusion

The serum level of vitamin D was found to be low, and the percentage of vitamin D deficiency was found to be high among children with asthma in Asia and Africa. Hence, early detection level of vitamin D and its deficiency is crucial to prevent the complications associated with vitamin D deficiency among children with asthma to decrease the frequency, attack, and exacerbation of asthma in Africa and Asia because together worsen one another.

## Data Availability

All data are available upon the request from the corresponding Author.

## Abbreviation:

JBI: Joanna Briggs Institute
PECO: Population, Exposure, Comparison, and Outcome
STATA: Statistical Analysis Software
